# Global research trends and hotspots in Parkinson’s disease psychosis: a 25-year bibliometric and visual analysis

**DOI:** 10.3389/fnagi.2024.1480234

**Published:** 2024-11-22

**Authors:** Jianhong Wu, Xin Jin, Weiming Xie, Liang Liu, Fei Wang, Ling Zhu, Yuan Shen, Linghe Qiu

**Affiliations:** ^1^Affiliated Mental Health Center of Jiangnan University, Wuxi Central Rehabilitation Hospital, Wuxi, Jiangsu, China; ^2^Northern Jiangsu People’s Hospital, Yangzhou, Jiangsu, China; ^3^Zhongnan Hospital of Wuhan University, Wuhan, Hubei, China; ^4^Jiangyin People's Hospital, Wuxi, Jiangsu, China

**Keywords:** Parkinson’s disease psychosis, PDP, bibliometric analysis, hotspots, global trends

## Abstract

**Background:**

Parkinson’s disease psychosis (PDP) is one of the most severe and disabling non-motor symptoms in the progression of Parkinson’s disease (PD), significantly impacting the prognosis of PD patients. In recent years, there has been an increase in literature on PDP. However, bibliometrics has rarely been applied to PDP research. This study provides an overview of the current state of PDP research and predicts future trends in this field.

**Methods:**

The literature search was conducted using the Web of Science Core Collection, with the search terms (Parkinson* AND (psychotic* OR hallucination* OR illusion* OR delusion* OR misperception* OR psychosis OR psychoses)). VOSviewer and CiteSpace software were employed to perform bibliometric analysis and visual representation of the search results.

**Results:**

A total of 603 articles were effectively included. Since 2017, there has been a significant upward trend in publications related to PDP. The United States, the United Kingdom, and Canada were the top three contributing countries in terms of publication volume, with France also having a strong influence in this field. *Movement Disorders* and King’s College London included and published the most articles on PDP. The paper titled “*Hallucinations in Parkinson’s Disease: Prevalence, Phenomenology, and Risk Factors*” received the highest number of citations and average citations. Cluster analysis results identified brain, prevalence, connectivity, and atypical antipsychotics as key hotspots in this field. High-frequency keywords were grouped into three themes: neurobiology, therapeutic strategies, and symptom research. Among them, pimavanserin, risk, and functional connectivity have been the most studied areas in the past 7 years and are likely to remain key topics in future research.

**Conclusion:**

Research on PDP has garnered increasing attention. This study visualizes PDP research over the past 25 years to analyze global hotspots and trends. It offers researchers a valuable perspective for identifying key topics and understanding research trajectories in this expanding field.

## 1 Introduction

Parkinson’s disease psychosis (PDP) is a common psychiatric manifestation in the natural course of Parkinson’s disease (PD) and is one of the most complex and disabling features among the various non-motor symptoms of PD. The hallmark characteristics include a spectrum of psychotic symptoms ranging from minor hallucinations (MHs) to widespread multimodal hallucinations and delusions (Ffytche et al., 2017a). More than 40% of patients with PD experience psychosis, and these symptoms tend to worsen as the disease progresses, leading to increased hospitalization rates (Rissardo et al., 2022), morbidity, and mortality (Eichel et al., 2022). PDP is one of the primary determinants of nursing home placement and caregiver burden, further complicating the management of PD (Angelopoulou et al., 2022). It severely impacts the quality of life for patients and caregivers, posing a significant challenge in the treatment of PD.

A 12-year longitudinal study on PDP populations revealed that patients with a higher age at PD onset and the presence of rapid eye movement (REM) sleep behavior disorder (RBD) have an increased risk of developing PDP. This pattern of risk factors, along with the concurrent development of psychiatric symptoms, disability, and dementia, places PDP within a symptom complex that suggests it is indicative of a malignant progression of PD (Forsaa et al., 2010). However, the exact neurobiological mechanisms remain unclear. Multiple brain structures and pathways are involved in the onset of PDP, including cortical and neuronal loss, gliosis, and Lewy body pathology (Fischer et al., 2021). It is currently established that the neurobiological mechanisms of PDP differ from those of other psychiatric disorders and present distinct clinical features (Weintraub and Mamikonyan, 2019). Therefore, integrating clinical assessment with the evaluation of PDP symptoms and mechanisms should be the foundation of ongoing research (Mangone et al., 2024). Factors such as visual processing disorders, neurotransmitter system and structural abnormalities, genetic factors, deep brain stimulation, and sleep disturbances are believed to be associated with the development of PDP (Zahodne and Fernandez, 2008). Hallucinations and delusions may result from the hyperactivation of pyramidal neurons in the visual cortex, leading to visual hallucinations (VHs), and the hyperactivation of the mesolimbic pathway (Cummings et al., 2022). Moreover, the treatment poses significant challenges. Controlling psychiatric symptoms without exacerbating motor dysfunction is crucial in the management of PDP (Cummings et al., 2014). Additionally, the visual disturbances associated with PDP are linked to a poorer prognosis (Naumann et al., 2021) and future cognitive decline (Powell et al., 2022). Early diagnosis and the prediction of risk factors are essential for delaying the onset of PDP. Therefore, a comprehensive review of PDP research is necessary.

With the increasing public awareness, the number of PDP-related studies has also been rising. Bibliometric analysis is a tool used to study the quantitative characteristics and distribution patterns of literature and related information. It combines methods from statistics, informatics, and social sciences to systematically analyze indicators such as the number of publications, citation counts, and author’s research output, thereby revealing trends and developments in scientific research (Donthu et al., 2021). Bibliometric analysis not only focuses on the impact of individual publications but also examines the overall performance of academic journals, authors, countries, and institutions, providing a deep understanding of research hotspots, knowledge structures, and academic communication. Core methods include citation analysis, collaboration network analysis, and topic modeling. These methods assist researchers in evaluating the academic impact of research outcomes, identifying key figures and institutions in the field, and providing a basis for scientific policy-making and resource allocation (Ellegaard and Wallin, 2015).

Currently, there are no bibliometric analysis papers specifically focused on the field of PDP. Therefore, this study conducts a detailed search of the Web of Science Core Collection (WoSCC) database and performs a bibliometric and visualization analysis of various aspects such as annual publication volume, international and institutional collaborations, journals, and keywords in this field. The aim is to explore the current state of research, key topics, and trends in PDP, providing valuable references for future studies.

## 2 Methods

### 2.1 Data source and search strategy

To ensure the credibility and comprehensive coverage of the data, the WoSCC was selected as the data source, covering the period from its inception to February 7, 2024. This included the Science Citation Index Expanded (SCI-EXPANDED) and the Social Sciences Citation Index (SSCI). The search terms used were related to PDP and were developed based on the Medical Subject Headings search strategy from PubMed. The details of the data source and search strategy are presented in Table 1.

**Table 1:** Summary of data source and search strategy.

Category	Specific standard requirements
Database	WoSCC
Citation Index	SCI-EXPANDED, SSCI
Period	the start of WoSCC to February 7, 2024
Language	English
Document types	Article, Review
Data extraction	Full record and cited references in plain text file
Searching strategies	#1: ((((((TS = (psychotic*)) OR TS = (hallucination*)) OR TS = (illusion*)) OR TS = (delusion*)) OR TS = (misperception*)) OR TS = (psychosis)) OR TS = (psychoses)
	#2: TS = (Parkinson*)
	#3: #2 AND #1

The search strategy identified papers that included these terms in the title, abstract, author keywords, or keywords plus. The data were then extracted from the selected publications, exported in the “Plain text file” format, and recorded as “Full record and cited references.” The language was restricted to English, and the publication type was limited to articles and reviews.

### 2.2 Data analysis

**Figure 1:**
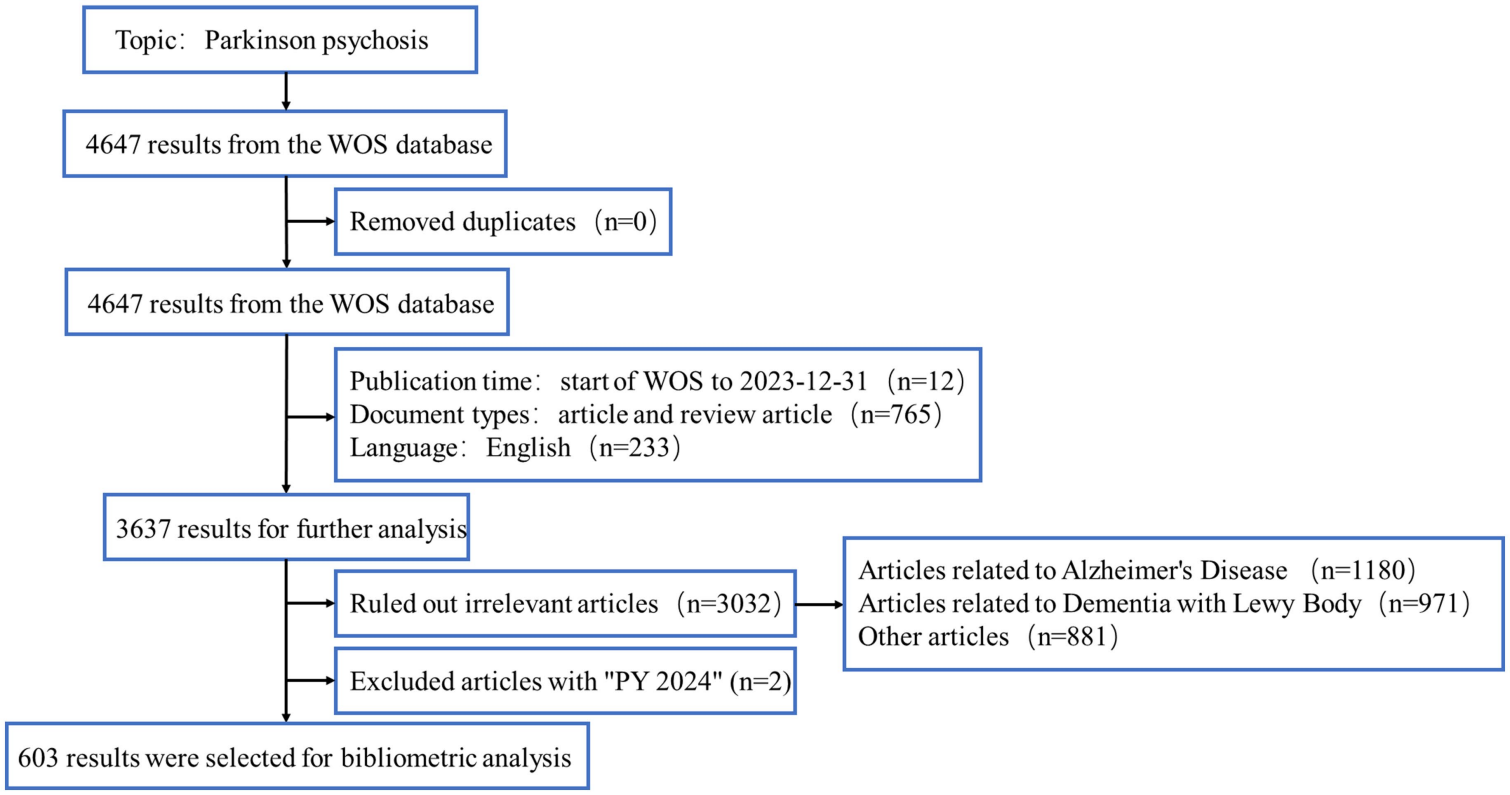
Flowchart of literature collection.

We reviewed the basic information of all documents, including titles, abstracts, and keywords, and consulted PubMed and Sci-Hub databases to complete any missing data. Articles with incomplete or irrelevant information were excluded. To ensure relevance, two authors independently reviewed and screened the papers. We excluded papers that focused primarily on other neurodegenerative diseases, such as Alzheimer’s Disease (AD) or dementia with Lewy bodies (DLB). However, studies that primarily focused on PDP but mentioned other diseases, such as AD or DLB, in a comparative or background context were retained. In cases where articles mention multiple neurodegenerative diseases, a detailed review was conducted to determine whether the focus of the study was primarily on PDP. Studies were retained if PDP was the central topic, even if other neurodegenerative diseases were discussed as part of the research or included in the discussion. Any disagreements were resolved through discussion. After the elimination process, a total of 603 documents remained, all of which were identified using CiteSpace. The flow chart of the data collection process is shown in Figure 1.

## 3 Results

### 3.1 Distribution of annual publications and citations

**Figure 2:**
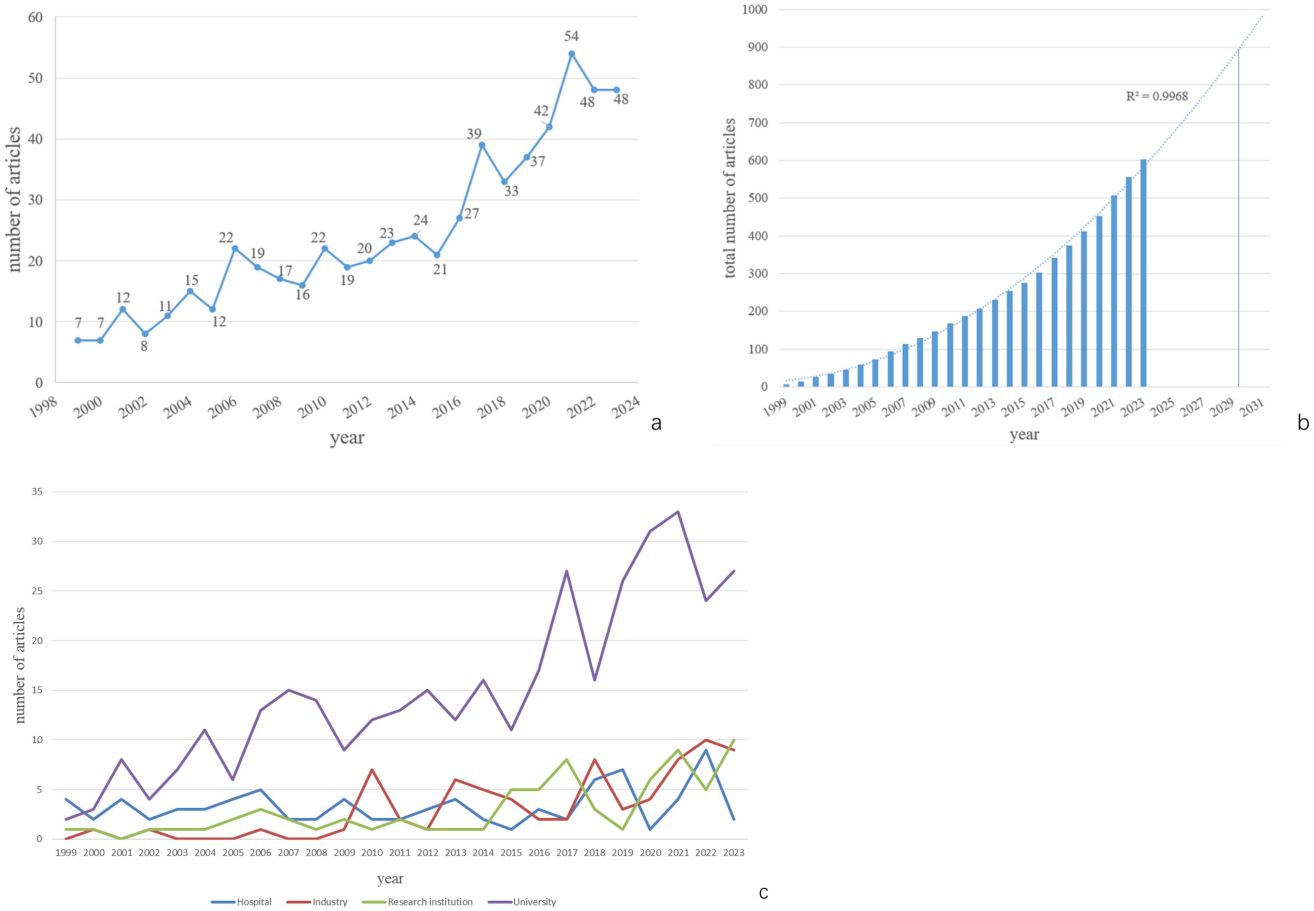
The number and trend of annual publications.

A total of 603 papers published from the inception of WoSCC to 2023 were used for data analysis. According to the trend analysis (Figure 2a), the number of publications exhibits a fluctuating upward trend. The number of published papers increased from 7 in 1999 to 48 in 2023. The growth in publications can be roughly divided into two phases: 1999–2016 and 2017–2023. From 1999 to 2016, the number of publications ranged from a few to several dozen per year. Starting in 2017, there was a dramatic increase in the number of publications, peaking in 2021. Although the second phase spans only 7 years, the number of published articles reached 301, surpassing the total from the first phase. Publications from the second phase account for 49.75% of all published literature. It is anticipated that the number of articles on PDP will increase to 900 by 2030 (Figure 2b).

By analyzing the funding sources or affiliations of the first authors, we categorized their affiliations into four groups: Hospital, Industry, Research Institution, and University. The number of publications for each year is shown in Figure 2c. It can be observed that University-affiliated publications were the most numerous overall. The growth in publication volume followed two stages similar to the overall trend. The publication volumes from Industry, Research Institutions, and Hospitals were generally comparable, with smaller fluctuations in annual output.

### 3.2 Analysis of countries

**Figure 3:**
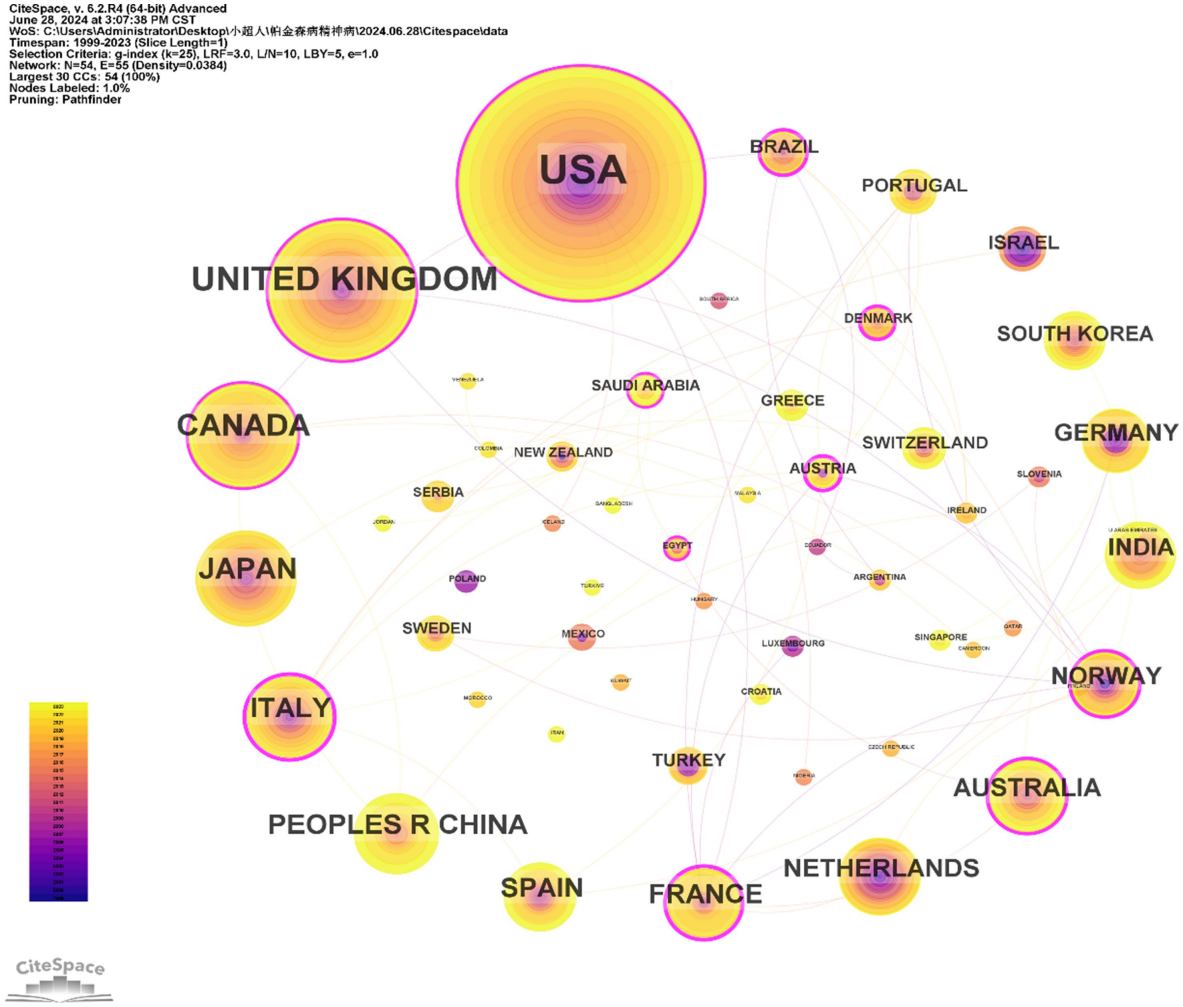
Collaborative research between countries on PDP.

The 603 papers were contributed by researchers from 54 different countries/regions. Figure 3 displays the distribution of publications by country/region. The top 10 countries with the highest number of publications were the USA (233), the United Kingdom (89), Canada (54), Japan (46), Italy (36), China (35), France (31), Spain (31), Netherlands (26), and Australia (24). Additionally, countries such as the USA, the United Kingdom, Canada, and Italy had a broad range of research activity over time, with early contributions (Figure 3). In contrast, China, Japan, and Spain have shown increased research activity in recent years. The USA also had the highest number of citations (9,483). As a metric for measuring the frequency of international collaboration, the size of centrality reflects the influence of a country within the global academic collaboration network. France and Norway, despite not being among the top in terms of publication volume, had the highest centrality and average citation rate, respectively. However, some prolific countries such as Japan, China, and Netherlands had a centrality of less than 0.01. The figure indicates that the connections between countries are not particularly close (Table 2).

**Table 2:** Top12 most productive countries on PDP.

Rank	Country	Documents	Citations	Averaged citations	Centrality	Year
1	USA	232	9,483	40.88	0.16	1999
2	United Kingdom	89	5,024	56.45	0.11	2001
3	Canada	54	1738	32.19	0.11	2001
4	Japan	46	1,042	22.65	0	2003
5	Italy	36	1,097	30.47	0.47	2001
6	China	35	538	15.37	0	2004
7	France	31	2,210	71.29	0.56	2000
8	Spain	31	1,216	39.23	0.05	2004
9	Netherlands	26	873	33.58	0	2001
10	Australia	24	765	31.88	0.25	2011
11	Norway	20	2,187	109.35	0.32	1999
12	Germany	19	591	31.11	0.06	1999

### 3.3 Analysis of journals

The number of publications by each journal provides an indication of its contribution to the dissemination of research on PDP. The 603 papers were published across 207 journals. The top 10 journals with the most publications on PDP are listed in Table 3. The journal *Movement Disorders* published the highest number of PDP-related papers (57; 9.45%; IF = 7.4), followed by *Parkinsonism & Related Disorders* (33; 5.47%; IF = 3.1) and *Clinical Neuropharmacology* (18; 2.99%; IF = 0.8). The 2023 impact factors (IF) of the top 10 journals ranged from 0.8 to 8.7, and these journals contributed 32.67% of the total publications. According to the Journal Citation Reports (JCR), 4 journals are classified as Q1, 5 as Q2, and 1 as Q4. Among the top ten journals, *Movement Disorders* had a significantly higher citation count (4,329), while *Neurology* had the highest average citation count.

**Table 3:** Top10 most productive journals on PDP.

Rank	Journal	Documents	Percent (%)	IF (2023)	JCR	Citations	Averaged citation
1	Movement Disorders	57	9.45	7.4	Q1	4,329	75.95
2	Parkinsonism & related disorders	33	5.47	3.1	Q2	1,030	31.21
3	Clinical Neuropharmacology	18	2.99	0.8	Q4	522	29.00
4	Neurology	16	2.65	7.7	Q1	1,612	100.75
5	Journal of Neurology	15	2.49	4.8	Q1	627	41.80
6	Journal of Geriatric Psychiatry and Neurology	12	1.99	2.9	Q2	225	18.75
7	Journal of Neurology Neurosurgery and Psychiatry	12	1.99	8.7	Q1	935	77.92
8	Journal of Parkinson’s Disease	12	1.99	4.2	Q2	173	14.42
9	Journal of Neural Transmission	11	1.82	3.2	Q2	321	29.18
10	Journal of Neuropsychiatry and Clinical Neurosciences	11	1.82	2.4	Q2	404	36.73

### 3.4 Analysis of institutions

Table 4 lists the most productive institutions in this field. The most prolific institution was King’s College London, with 27 publications, followed by ACADIA Pharmaceuticals Inc. with 23 publications, and Rush University with 22 publications. Among the top ten institutions, most are located in Canada, USA, and the United Kingdom. Rush University has the highest number of citations and average citations, indicating its significant influence in this field.

**Table 4:** Top10 most productive organizations on PDP.

Rank	Organization	Country	Documents	Citations	Averaged citation
1	King’s College London	United Kingdom	27	1,414	52.37
2	ACADIA Pharmaceuticals Inc	USA	23	420	18.26
3	Rush University	USA	22	2,732	124.18
4	Brown University	USA	20	1,341	67.05
5	McGill University	Canada	20	274	13.70
6	University of Toronto	Canada	20	982	49.10
7	University of Pennsylvania	USA	18	811	45.06
8	Université de Montréal	Canada	17	265	15.59
9	University College London	United Kingdom	16	1,071	66.94
10	The University of Sydney	Australia	16	556	34.75

**Figure 4:**
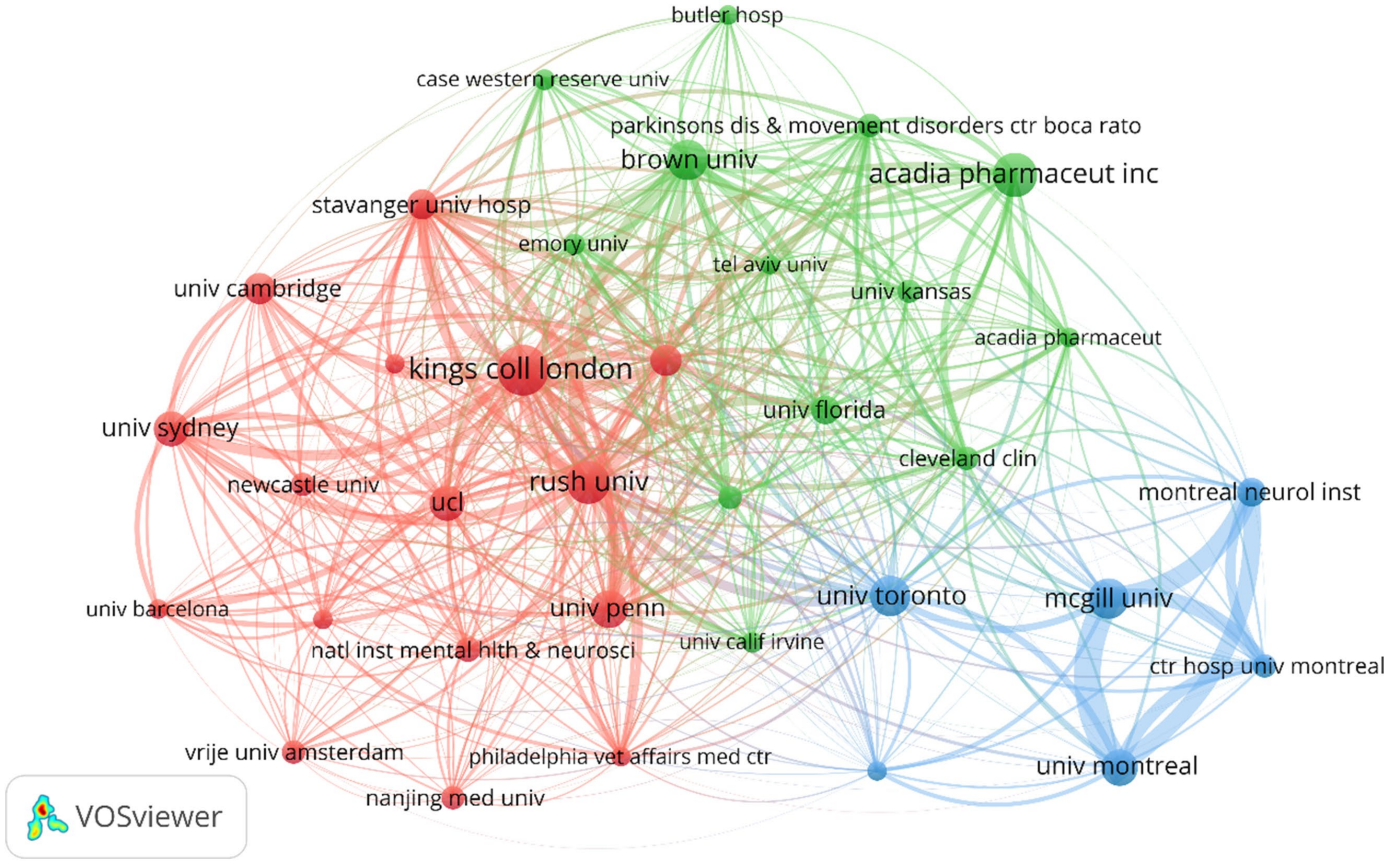
Visualization of the collaboration network of institutions in PDP.

Figure 4 illustrates the partnerships between different institutions. Colors represent different clusters, nodes indicate individual institutions, and lines represent connections between institutions. The red cluster includes institutions with extensive research and collaboration on PDP symptoms and mechanisms. The green cluster involves cross-research on PDP drug therapies, including 5-HT2A inverse agonists like pimavanserin and other atypical antipsychotics (AAPs), and includes pharmaceutical companies and universities. The blue cluster shows active collaboration among Canadian institutions, particularly those in Montreal and its affiliated hospitals, the University of Toronto, and McGill University. Additionally, King’s College London, ACADIA Pharmaceuticals Inc., and McGill University play significant roles within their respective collaboration clusters. The dense connections between nodes such as the University of Pennsylvania, Rush University, and Johns Hopkins University indicate frequent collaboration among these institutions.

### 3.5 Analysis of the 10 most-cited article

The number of citations for research publications is a standard measure of academic impact. Table 5 lists the top 10 most-cited papers, with citations ranging from 241 to 719. Among these, 8 papers were published in Q1 journals. The most-cited paper was by G. Fénelon, published in 2000, titled “*Hallucinations in Parkinson’s Disease: Prevalence, Phenomenology and Risk Factors*” in *Brain* (IF = 10.6; Q1), with 719 citations.

**Table 5:** Top 10 most-cited articles.

Rank	Author, Year	Title	Journal	IF (2023)	JCR	Citations	Average Citation
1	G Fénelon, 2000	Hallucinations in Parkinson’s disease: Prevalence, phenomenology and risk factors	Brain	10.6	Q1	719	29.96
2	Jeffrey Cummings, 2014	Pimavanserin for patients with Parkinson’s disease psychosis: a randomized, placebo-controlled phase 3 trial	Lancet	98.4	Q1	438	43.8
3	D Aarsland, 1999	Mental symptoms in Parkinson’s disease are important contributors to caregiver distress	Int J Geriatr Psychiatry	3.6	Q1	340	13.6
4	Bernard Ravina, 2007	Diagnostic criteria for psychosis in Parkinson’s disease: Report of an NINDS, NIMH work group	Mov Disord	7.4	Q1	331	19.47
5	J M Miyasaki, 2006	Practice Parameter: evaluation and treatment of depression, psychosis, and dementia in Parkinson disease (an evidence-based review): report of the Quality Standards Subcommittee of the American Academy of Neurology	Neurology	7.7	Q1	310	17.22
6	D Aarsland, 1999	Prevalence and Clinical Correlates of Psychotic Symptoms in Parkinson Disease A Community-Based Study	Arch Neurol	N/A	N/A	285	11.4
7	Neil K Archibald, 2009	The retina in Parkinson’s disease	Brain	10.6	Q1	269	17.93
8	S Holroyd, 2001	Prospective study of hallucinations and delusions in Parkinson’s disease	J Neurol Neurosurg Psychiatry	8.7	Q1	267	11.61
9	Elin B Forsaa, 2010	A 12-Year Population-Based Study of Psychosis in Parkinson Disease	Arch Neurol	N/A	N/A	258	18.43
10	Rimona S Weil, 2016	Visual dysfunction in Parkinson’s disease	Brain	10.6	Q1	241	30.13

Among the top 10 most-cited papers, only two were published in the past decade. The paper by Jeffrey Cummings, published in 2014, titled “*Pimavanserin for Patients with Parkinson’s Disease Psychosis: A Randomized, Placebo-Controlled Phase 3 Trial*” in *Lancet* (IF = 98.4; Q1), had the highest average annual citations in the field. These highly cited papers primarily focused on symptoms, mechanisms, prevalence, diagnosis, treatment, and the evaluation of therapeutic efficacy of PDP.

### 3.6 Analysis of keywords

#### 3.6.1 Keywords co-occurrence networks

**Figure 5:**
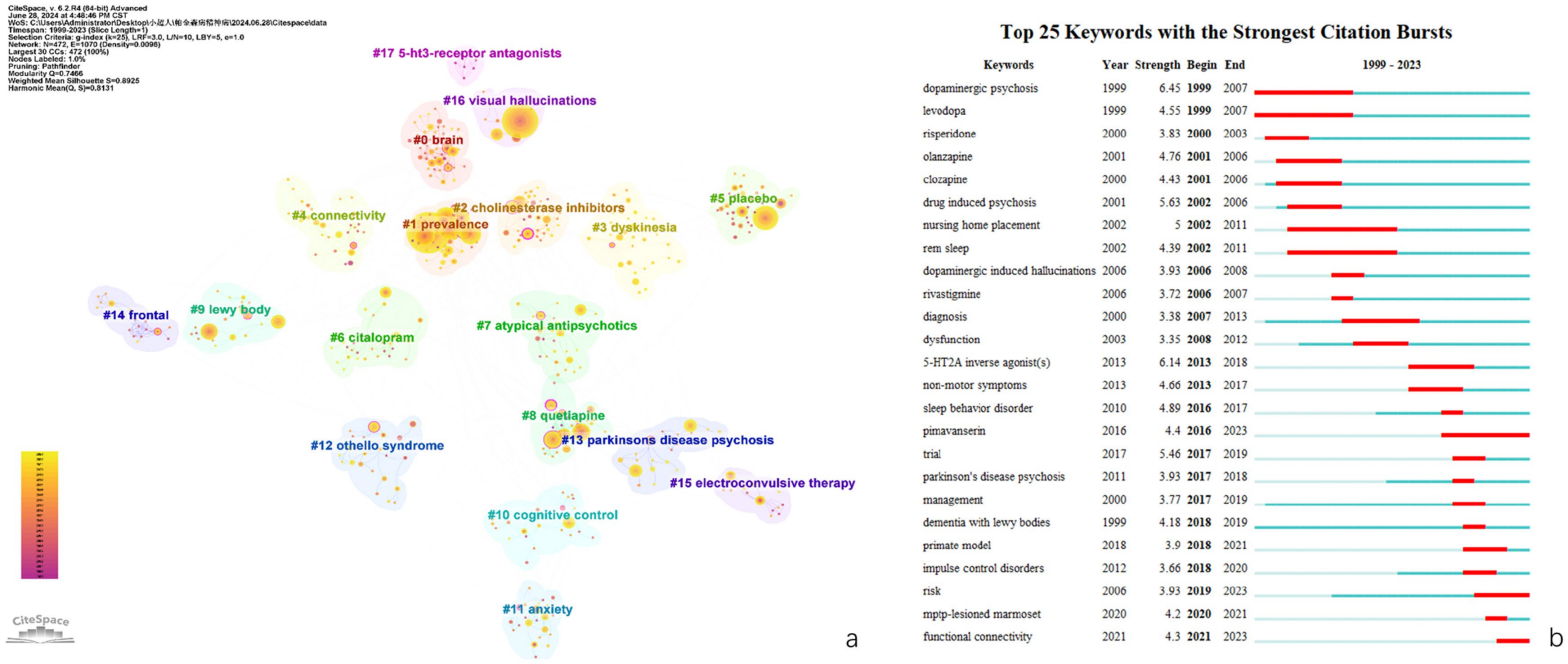
Network visualization map of keywords co-occurrence (a) and top 25 keywords with the strongest citation bursts (b).

Figure 5a displays a keyword cluster analysis. Each cluster contains numerous keyword points, with the most weighted keywords serving as cluster labels. Using the Log-likelihood Ratio (LLR) algorithm, current research can be divided into 17 clusters (Q = 0.7466, S = 0.8925). After removing irrelevant topics, the clusters are defined in Table 6.

**Table 6:** Clustering labels and directions.

Cluster	Label (LLR)	Direction
#0	Brain	Investigates structural and functional changes in the brain related to PDP, involving neuroimaging and pathology studies.
#1	Prevalence	Focuses on the epidemiology of PDP, including its incidence and prevalence.
#2	Cholinesterase Inhibitors	Examines the therapeutic effects and mechanisms of cholinesterase inhibitors in PDP.
#3	Dyskinesia	Addresses motor disorders in Parkinson’s disease.
#4	Connectivity	Explores changes in brain network connectivity in PDP and their impact on symptoms.
#7	Atypical Antipsychotics	Investigates the use and efficacy of AAPs in PDP treatment.
#8	Quetiapine	Studies the efficacy and safety of quetiapine in PDP.
#9	Lewy Body	Researches the role and pathological mechanisms of Lewy body deposits in PDP.
#10	Cognitive Control	Examines the relationship between cognitive control and symptoms of PDP, and its impact on daily life.
#11	Anxiety	Investigates anxiety symptoms as predictors of PDP.
#12	Othello Syndrome	Studies jealousy delusions (Othello syndrome) in PDP and their pathological mechanisms.
#13	Parkinson’s Disease Psychosis	The core theme focusing on symptoms, diagnosis, pathological mechanisms, and treatment methods of PDP.
#14	Frontal	Investigates the role and functional changes of the frontal lobe in PDP.
#15	Electroconvulsive Therapy	Explores the application and efficacy of electroconvulsive therapy in PDP.
#16	Visual Hallucinations	Studies the mechanisms and management of visual hallucinations in PDP.
#17	5-HT3 Receptor Antagonists	Researches the potential role of 5-HT3 receptor antagonists in PDP treatment.

These clusters represent different directions in PDP research, covering a broad range from basic science to clinical treatment. They provide a better understanding of the multifaceted nature of PDP and potential treatment approaches.

#### 3.6.2 Keywords with the strongest citation bursts

Table 7 displays the most frequently occurring keywords in this study. Besides “Parkinson’s disease” and “psychosis,” the top three keywords were “visual hallucinations” (254), “dementia” (191), and “hallucinations” (121). Figure 5b illustrates the top 25 keywords with the most significant citation bursts since 1999. The length of the red bars represents the duration of the research frontier, while the intensity of the burst reflects the magnitude of the citation increase. The keywords “dopaminergic psychosis,” “drug-induced psychosis,” and “olanzapine” experienced the strongest early bursts. After 2007, the keywords “5-HT2A inverse agonist(s),” “trial,” and “sleep behavior disorder” had the most substantial citation bursts. The latest research frontiers included “pimavanserin,” “risk,” and “functional connectivity.”

**Table 7:** Top 22 keywords in PDP.

Rank	Keyword	Count	Centrality	Year	Rank	Keyword	Count	Centrality	Year
1	Parkinson’s disease	387	0.01	1999	12	Clozapine	74	0.05	2000
2	Visual hallucinations	254	0.04	1999	13	Cognitive dysfunction	72	0.20	2000
3	Dementia	191	0.05	1999	14	Alzheimer’s disease	67	0.09	1999
4	Psychosis	159	0.03	1999	15	Diagnosis	48	0.11	2000
5	Hallucinations	121	0.01	2000	16	Quetiapine	48	0.04	1999
6	Symptoms	110	0.04	2001	17	Non-motor symptoms	42	0	2013
7	Drug induced psychosis	99	0	2001	18	Neuropsychiatric symptoms	40	0.01	2005
8	Risk factors	98	0.01	2002	19	Dementia with Lewy bodies	39	0.21	1999
9	Prevalence	98	0.02	2001	20	Nursing home placement	39	0.05	2002
10	Lewy body	86	0.03	2001	21	Atypical antipsychotic(s)	38	0.16	2000
11	Double blind	81	0.13	1999	22	Parkinson’s disease psychosis	38	0.03	2011

## 4 Discussion

### 4.1 General information

This study is the first comprehensive bibliometric analysis of the research trends in PDP. By analyzing 603 publications from 1999 to 2023, it reveals a rapid growth trend in PDP research, particularly over the past 7 years. This trend indicates that PDP has become an emerging and popular research topic. Key milestone articles have significantly influenced subsequent research directions. For example, the provisional diagnostic criteria for PDP established by the National Institute of Neurological Disorders and Stroke (NINDS) and the National Institute of Mental Health (NIMH) in 2007 summarized specific definitions of PDP-related symptoms, marking the onset of the literature’s explosive growth (Ravina et al., 2007). Fénelon et al.’s detailed exploration of PDP-related hallucinations, particularly identifying three potential risk factors and emphasizing that simple side effects of dopaminergic treatments cannot account for all hallucinations, expanded the direction of PDP research (Fénelon et al., 2000). Additionally, Cummings et al.’s randomized, double-blind, placebo-controlled study of pimavanserin has driven the development of new mechanism-based drugs for PDP (Cummings et al., 2014). These important papers not only provide valuable insights into the various manifestations and mechanisms of PDP but also offer theoretical support for future, more in-depth research.

Under the influence of the PDP diagnostic criteria proposed by the NINDS/NIMH workgroup (Ravina et al., 2007), research findings have been published across multiple countries. The USA was the most productive country in terms of research output, but the outcomes did not fully align with centrality, indicating that the USA has a significant number of high-quality independent studies (Mack et al., 2012; Holroyd et al., 2001). Although the USA publications were highly cited domestically, international collaboration and network connections were relatively limited. France and Italy, despite having fewer publications, exhibit high centrality, suggesting that their research had significant academic impact and was widely cited and recognized internationally (Fénelon et al., 2000). Norway, due to its early contributions in epidemiological assessments and caregiver research on PDP, had filled gaps in this field and received considerable attention, reflected in the highest average citations (Forsaa et al., 2010; Aarsland et al., 1999a,b). Notably, Japan, China, and Netherlands had low centrality, which may indicate that research in these countries was primarily focused on domestic issues with limited international collaboration. These countries had distinct regional characteristics, with research concentrated in specific areas: China focused on MHs in PDP (Zhong et al., 2021a, 2022); Japan on VHs (Matsui et al., 2006) and familial gene mutations (Yoshino et al., 2017) in PDP; and the Netherlands primarily on VHs (Meppelink et al., 2009; Janzen et al., 2012).

Aside from differences in publication quantity and centrality, research on PDP exhibits significant variations in focus across different countries. These differences are likely influenced by factors such as economic development, research investment, healthcare resource allocation, and cultural background. In developed countries like USA and some European nations, PDP research was relatively mature and advanced. These countries benefit from substantial funding and state-of-the-art research facilities, enabling complex neuroimaging studies and clinical trials for new medications (Cummings et al., 2014). In contrast, some developing countries may focus more on epidemiological characteristics and clinical management strategies for PDP. These nations may lack the necessary research funding and equipment for in-depth mechanistic studies but have accumulated valuable experience in patient management and care (Zhong et al., 2021a). Additionally, some European countries, such as Norway, emphasized not only biomedical research but also the psychological well-being and social support systems for caregivers (Aarsland et al., 1999b). The fact that nearly hundreds of countries contributed to the field of PDP research indicates its broad global scope. However, future research will benefit from more extensive international collaborative efforts.

Journal analysis can assist researchers in selecting appropriate journals for their work. Different journals, based on their focus, target audience, and academic impact, exhibit unique characteristics in literature inclusion and research emphasis. High-impact journals in medicine and neuroscience, such as *Movement Disorders*, typically published research with significant clinical relevance and innovation. *Movement Disorders* frequently featured studies on PDP-related VHs (Nagano-Saito et al., 2004; Watanabe et al., 2013), neurophysiology of brain regions (Meppelink et al., 2011), brain networks (Baik et al., 2024), and molecular mechanisms (Camicioli et al., 2005), as well as articles evaluating the efficacy (Juncos et al., 2004; Mohr et al., 2000; Espay et al., 2018) and safety (Friedman et al., 2006) of drugs or treatments. The journal also emphasizes the assessment of PDP scales (Virués-Ortega et al., 2010; Voss et al., 2010) and the establishment of diagnostic criteria (Ravina et al., 2007). In contrast, *Parkinsonism & Related Disorders* focused more on the symptomatic features (Barrett et al., 2017; Pachi et al., 2021) and social dimensions (Wetmore et al., 2019) of PDP, including assessments of scales (Voss et al., 2013) and treatment strategies (Weintraub et al., 2024). Interdisciplinary journals, such as the *Journal of Neurology* and the *Journal of Geriatric Psychiatry and Neurology*, provided platforms for a variety of research types and methods, including basic research (Carter and Ffytche, 2015), clinical studies (Bugalho et al., 2012; Schneider et al., 2024), and review articles (Toh et al., 2023). Some journals may be more specialized in basic research, like *Neurology*, which often published studies on the neurobiological mechanisms of PDP (Zarkali et al., 2020a). Others, such as *Clinical Neuropharmacology*, focused more on clinical pharmacology and therapeutic interventions, publishing research on drug efficacy and side effects (Klein et al., 2006). The fact that fewer than one-third of the publications were distributed across the top ten most active journals indicates a broad distribution across various journals. This dispersion likely reflects the interdisciplinary nature of PDP research, which spans fields such as neurobiology, psychiatry, biochemistry, molecular biology, clinical medicine, and pharmacology.

The research differences between institutions reflect the diversity and complexity of studies in the PDP field. Institutional research focuses are often influenced by resources, team expertise, and regional needs. King’s College London and ACADIA Pharmaceuticals Inc. were pivotal nodes in the co-occurrence network, collaborating with various institutions. King’s College London has made significant contributions to understanding hallucinations of PDP, exploring its prevalence (Gibson et al., 2013), characteristics (Ffytche et al., 2017b), potential mechanisms (Whitehead et al., 2008; Pisani et al., 2023), treatment strategies (Shotbolt et al., 2009), and prognosis (Barnes and David, 2001). They emphasized that VHs in PDP patients might stem from misprocessing environmental stimuli (Barnes et al., 2003), potentially linked to abnormalities in the default mode network (DMN) (Yao et al., 2014). Their large-scale data analyses have also highlighted the epidemiological features of PDP (Gibson et al., 2013), informing public health policies. ACADIA Pharmaceuticals Inc., on the other hand, focused on pharmacological treatment strategies for PDP. In collaboration with King’s College London, they conducted a randomized, placebo-controlled Phase 3 trial on pimavanserin (Cummings et al., 2014), identifying it as a selective 5-HT2A receptor inverse agonist beneficial for PDP treatment (Hacksell et al., 2014). Compared to other AAPs, pimavanserin significantly improved PDP symptoms, such as those measured by the Scale for the Assessment of Positive Symptoms (SAPS) and the Unified Parkinson’s Disease Rating Scale (UPDRS) Parts II and III, with fewer side effects (Meltzer et al., 2010). Their collaboration with Brown University further explored drug efficacy and safety, proposing new strategies for optimizing PDP treatment. ACADIA Pharmaceuticals Inc. also highlighted pimavanserin’s lower all-cause hospitalization and psychiatric hospitalization rates (Rajagopalan et al., 2023a) and contributed to pediatric dosing studies (Darwish et al., 2023), expanding research to cover a broader age range.

The collaboration network illustrates a trend of homogeneity, reflecting close cooperation and research clustering among institutions within the same region or focusing on similar research themes. For instance, King’s College London and the University of Pennsylvania have jointly researched the spectrum of psychiatric symptoms in PDP (Ffytche et al., 2017a), including studies on the relationship between cognitive and executive deficits and quality of life deterioration in PDP patients (Pisani et al., 2024). Institutions such as Rush University have made significant contributions to epidemiological studies on PDP, including age of onset, risk factors (Forsaa et al., 2010), positive rates of scales (Fernandez et al., 2008), pathophysiology of hallucinations (Diederich et al., 2005), and treatments (Diederich et al., 2009). Another important collaborative cluster is found within Canadian institutions, including Montreal, McGill University, and the University of Toronto. These institutions have made notable contributions to PDP mechanism research, from neurobiological foundational studies to clinical treatment trials. They have played a crucial role in studying 5-HT2A receptor signaling (Ballanger et al., 2010) and evaluating clinical research drugs for treating psychosis in parkinsonian marmoset (Hamadjida et al., 2018; Frouni et al., 2019). Rush University has also established diagnostic criteria for PDP, laying the groundwork for the explosion of subsequent literature, including collaborations with Brown University (Ravina et al., 2007). Additionally, Rush University, along with the University of Toronto and the University of Pennsylvania, has published practice guidelines for PDP treatment (Miyasaki et al., 2006), providing a basis for clinical practice. Collaboration among different research institutions has been crucial in advancing PDP research, addressing resource limitations in individual countries, and facilitating cross-validation and application of research findings. However, institutions with lower centrality indicate areas where future research could benefit from enhanced collaboration between core institutions in different countries.

The most cited papers in the field reflect the most valuable and impactful discoveries, highlighting key research hotspots and trends. The article “*Hallucinations in Parkinson’s disease: Prevalence, phenomenology and risk factors*” published in *Brain*, was the most cited article (Fénelon et al., 2000). This study addressed the phenomenology, prevalence, and risk factors of PDP hallucinations, identifying three potential risk factors: severe cognitive impairment, daytime sleepiness, and the chronicity of PD. The study also emphasized that simple side effects of dopaminergic treatments are insufficient to explain all VHs and notes that the overall prevalence of PDP with MHs is significantly higher than previously reported. The second most cited paper was Cummings et al.’s 2014 study “*Pimavanserin for patients with Parkinson’s disease psychosis: a randomized, placebo-controlled phase 3 trial*” published in *Lancet* (Cummings et al., 2014). This research, a 6-week randomized, double-blind, placebo-controlled trial, found that pimavanserin could be beneficial for PDP patients. In recent years, research into PDP medications has been a hot topic, particularly focusing on 5-HT2A inverse agonists like pimavanserin. Interestingly, pimavanserin remains highly relevant in our keyword co-occurrence analysis, reflecting ongoing interest in developing treatment strategies. These highly cited works underscore significant developments in understanding PDP and advancing therapeutic approaches, revealing ongoing trends and priorities in the field.

### 4.2 Hotspots and frontiers

PDP has garnered significant attention in recent years as a crucial clinical feature of PD. Hotspot analysis indicates that research related to PDP has primarily focused on several key areas: neurobiology (such as brain, functional connectivity, and Lewy bodies), therapeutic strategies (including cholinesterase inhibitors, AAPs, pimavanserin, and electroconvulsive therapy (ECT)), and symptom research (such as VHs, dyskinesia, Othello syndrome, and cognitive control). Additionally, research has covered diagnosis, underlying mechanisms, and the impact on caregivers. Among these, symptom research has consistently been a focal point and a core content area in PDP studies. In recent years, there has been a shift in research focus toward identifying early risk factors for PDP (Knolle et al., 2023) and mitigating its impact on daily life. Understanding how symptoms such as depression and anxiety exacerbate hallucinations and delusions has also emerged as a significant research interest (Pachi et al., 2023). Moving forward, it is expected that more studies will concentrate on the early identification of symptoms and the development of personalized management strategies for PDP. This ongoing shift in research priorities reflects a broader trend toward enhancing the quality of life for PDP patients by addressing the complex interplay of symptoms and refining therapeutic approaches to manage the condition more effectively.

In 2007, the concept of PDP was first introduced into international research. Prior to 2007, although the term “Parkinson’s Disease Psychosis” had not been widely adopted, symptoms such as hallucinations, delusions, and other psychotic features were already recognized in patients with Parkinson’s disease (Thanvi et al., 2005). These symptoms were often referred to as neuropsychiatric or behavioral complications associated with Parkinson’s disease, without a distinct label (Ferreri et al., 2006). Studies from the 1990s and early 2000s often focused on these psychotic symptoms, but the terminology and diagnostic criteria varied significantly across the literature (Doraiswamy et al., 1995; Williams-Gray et al., 2006). The introduction of the concept of “Parkinson’s Disease Psychosis” in 2007 helped standardize the terminology and diagnosis of these symptoms, leading to a more structured approach to research and clinical management (Ravina et al., 2007). This included unifying the previously recognized various symptoms of PDP into a framework of Parkinson’s disease psychosis spectrum symptoms. It also involved identifying a set of distinct clinical features specific to PDP, rather than shared with other psychotic syndromes (Ffytche et al., 2017a). This shift allowed for clearer criteria in identifying psychosis specifically related to Parkinson’s disease, as distinct from other neuropsychiatric conditions. Since then, the field has seen significant advancements in understanding the underlying mechanisms of PDP and the development of targeted treatment strategies. As a distinct clinical entity, research on PDP has become more focused, leading to a clearer understanding of its prevalence, risk factors, and management (Chang and Fox, 2016).

Under the new definition, studies on PDP symptoms have evolved from focusing on individual symptoms to examining a spectrum of continuous symptoms (Ffytche et al., 2017a). This spectrum includes everything from “minor” hallucinations to well-structured hallucinations and delusions. The most common and early psychiatric symptoms in PDP patients are MHs, which include visual illusions, passage hallucinations, and presence hallucinations (Ffytche et al., 2017a). These symptoms may even precede the onset of motor symptoms. Illusions are the most prevalent type of MHs, affecting more than one-third of PD patients. The most commonly reported types include complex visual illusions, kinetopsia, and pelopsia (Jucevičiūtė et al., 2024). Other associated conditions include isolated diplopia (Nebe and Ebersbach, 2007) and spatial misjudgment. MHs typically occur before the onset of well-structured VHs and are accompanied by other cumulative non-motor symptoms such as RBD (Jiang et al., 2023), cognitive impairment (Pagonabarraga et al., 2016; Pacchetti et al., 2005), and depression (Pachi et al., 2023). There are clinical differences in the characteristics of MHs between different phenotypes. For example, patients with the postural instability gait difficulty (PIGD) phenotype have a higher incidence of MHs, particularly visual illusions, a shorter latency period, earlier onset, and worse prognosis compared to those with the tremor-dominant phenotype (Wang et al., 2023).

VHs is the main clinical symptom of PDP patients, which manifest as complex images frequently involving people or animals. These visual images can be dynamic and tend to occur in low-stimulus environments, typically when the individual is alone in a quiet setting. These hallucinations may occur several times a day during the early stages of PDP, lasting from a few seconds to several minutes. The progression of cognitive decline and loss of insight often parallels the advancement of these symptoms. As the disease progresses, patients may lose the ability to recognize hallucinations as unreal, leading to the emergence of multimodal hallucinations and delusions, a condition referred to as “malignant hallucinations.” These malignant hallucinations are disabling, often accompanied by paranoid thoughts characterized by suspicion, blame, and unkemptness (Ffytche et al., 2017a). Multimodal hallucinations in PDP extend beyond visual experiences to include non-VHs such as auditory hallucinations (Clark, 1998), tactile hallucinations, and olfactory hallucinations (Bannier et al., 2012), with auditory hallucinations often occurring alongside visual ones. Once any form of hallucination appears, it tends to persist intermittently. Studies utilizing polysomnography have revealed that Parkinson’s patients experiencing hallucinations typically have poorer sleep quality and altered sleep architecture (Gu et al., 2022).

The keyword “Othello syndrome” refers to a type of delusional disorder that can occur in the later stages of PDP (Cannas et al., 2009). Delusions are irrational, unfounded, and often impossible beliefs that patients hold with conviction despite contrary evidence. In PDP, the occurrence of delusions is less frequent than hallucinations, but they tend to be more severe and disruptive, causing significant negative impacts on both patients and their families. Delusions in PDP are often accompanied by visual and auditory hallucinations, and compared to patients who experience only hallucinations, those with delusions are more likely to be younger PD patients and to exhibit aggressive and violent behaviors (Laurell et al., 2023). The delusions in PDP are predominantly of a paranoid nature, typically presenting as mixed-type delusions, with isolated delusions being less common. These delusions manifest in various forms, including guilt, grandeur, religious themes, persecution, jealousy, and theft (Factor et al., 2014), with considerable heterogeneity among cases (Warren et al., 2018). “Othello syndrome,” named after the character Othello from Shakespeare’s play Othello, describes a severe form of jealousy, often accompanied by paranoia and delusions, typically involving a strong belief in a partner’s infidelity. Research suggests that Othello syndrome may be associated with increased dopamine levels in the mesolimbic pathway (Cannas et al., 2006), particularly related to dopamine receptor hypersensitivity and neurotransmitter imbalances (Wolters, 1999).

The keyword “dementia with Lewy bodies” (DLB) refers to a disease that closely resembles the symptoms of PDP, especially when dementia symptoms manifest in PDP patients. During the literature screening process, a significant number of publications related to DLB were excluded. In the analysis of Keywords with the Strongest Citation Bursts, DLB consistently appeared with low citation burst frequency, highlighting the clinical and research challenges in differentiating between DLB and PD, as well as the diagnostic difficulties associated with both conditions. DLB is a neurodegenerative disease characterized pathologically by Lewy bodies and clinically by fluctuating progressive dementia, persistent attention deficits, visuospatial impairments, persistent complex VHs, and spontaneous parkinsonism (McKeith et al., 2017). VHs is a core feature of DLB diagnosis and is also a typical feature of PDP (Zhong et al., 2021b), making the two conditions easily confusable, especially since both exhibit parkinsonian symptoms. However, the VHs in DLB are generally more specific compared to those in PD. Most DLB patients experience true VHs that are often vivid and well-formed. The hallucinated objects are frequently familiar figures, such as people or animals, which are usually animated, speaking, or making sounds. Occasionally, these hallucinations may be distorted or grotesque. These hallucinations typically present as early symptoms and tend to respond poorly to pharmacological treatments (Ferman et al., 2011). In contrast, VHs in PDP patients often co-occur with other forms of hallucinations and typically emerge in the later stages of PD. These symptoms can be partially alleviated through medication (Cummings et al., 2014). Another distinguishing factor is that DLB generally begins with early cognitive impairment that precedes motor dysfunction, while PDP begins with motor dysfunction and later develops psychiatric spectrum symptoms, with cognitive impairment not being a primary diagnostic criterion (Jellinger and Korczyn, 2018).

Therefore, the keyword “diagnosis” highlights the critical role of diagnosing PDP in understanding and managing the condition. The diagnostic criteria established by the NINDS/NIMH have provided a vital theoretical and practical foundation for identifying PDP (Ravina et al., 2007). According to these criteria, PDP requires the presence of one or more of the following symptoms: hallucinations, illusions, false perceptions, or delusions, which must persist for more than 1 month or recur frequently, while ruling out other causes such as DLB and psychiatric disorders. Specific scales can enhance the reporting of PDP symptoms, allowing for the early detection of these symptoms in the disease’s course (Greger et al., 2022). The Movement Disorder Society (MDS) UPDRS (MDS-UPDRS) Part I is useful for assessing PDP at various stages (Goetz et al., 2008). Other scales, such as the Parkinson Psychosis Questionnaire (PPQ) (Brandstaedter et al., 2005), the SAPS for Parkinson’s Disease Psychosis (SAPS-PD) (Schubmehl and Sussman, 2018) and its modified version (Kulick et al., 2018), the Scale for Evaluation of Neuropsychiatric Disorders in Parkinson’s Disease (SEND-PD) (Martinez-Martin et al., 2012), the Parkinson Psychosis Rating Scale (PPRS) (Friedberg et al., 1998), and the Self-Administered Screening Questionnaire for Parkinson’s Disease-Associated Psychosis (SASPAP) (Koneru et al., 2023), can be specifically applied to evaluate PDP. For screening PDP, the UPDRS is first used to screen for psychiatric disorders, followed by assessments like SAPS to evaluate the severity of PDP. These scales can be combined for more comprehensive evaluation (Fernandez et al., 2008).

The clustering of keywords like “brain,” “frontal,” “connectivity,” and “Lewy body” indicates that research into the mechanisms of PDP is a major focus and is crucial for developing precise and early diagnostic methods. A Mendelian randomization study supports the inherent nature of PDP symptoms (Kim et al., 2021). PDP is associated with underlying neurodegenerative processes, where cortical and neuronal loss, gliosis, and Lewy body pathology collectively explain the neuropsychiatric symptoms (Fischer et al., 2021). PDP patients exhibit extensive grey matter volume loss, affecting at least 14 different regions (Ffytche et al., 2017a), particularly within the visual processing pathways (ventral and dorsal thalamus) (Pagonabarraga et al., 2016), pedunculopontine nucleus, and inferior frontal gyrus (Broadstock et al., 2014), as well as other related anatomical structures, including the auditory cortex (Lenka et al., 2015). Frontal lobe dysfunction is significant in PDP patients (Thota et al., 2017), with a noticeable reduction in overall hippocampal volume, hippocampal subfield atrophy, and a widening of the bilateral hippocampal fissure (Lenka et al., 2018). Neuropsychological testing has shown that the gradual loss of insight reflects the progression from frontal-striatal network dysfunction to posterior cortical damage (Wang et al., 2018). Key factors contributing to VHs include reduced fiber bundle cross section within the splenium of the corpus callosum, specific white matter tract degeneration (Zarkali et al., 2020a), and elongated visual evoked potentials latency (Matsui et al., 2005). Additionally, areas such as the parahippocampal gyrus, amygdala, frontal lobe, middle temporal gyrus, entorhinal cortex, and anterior cingulate cortex exhibit higher pathological Lewy body density (Gallagher et al., 2011), which correlates with the onset of PDP and VHs (Williams and Lees, 2005). The spectrum of PDP symptoms reflects the Braak progression of Lewy body pathology from the brainstem to the forebrain systems (Braak et al., 2003), highlighting the widespread impact of brainstem lesions on subcortical and cortical motor as well as oculomotor control networks, including the visual parietal lobe.

The keyword “visual hallucinations” highlights the critical importance of this symptom in PDP, aligning with bibliometric findings on VHs (Zhong et al., 2021b). Various models have been proposed to explain VHs, each reflecting different understandings of brain function (Collerton et al., 2023). Functional magnetic resonance imaging (fMRI) and positron emission tomography (PET) studies indicate that brain activity patterns in PDP patients significantly differ from those in non-psychotic patients, especially regarding functional abnormalities in the prefrontal cortex and visual cortex. Some researchers suggest that hallucinations arise from altered effects within different brain networks, with changes in functional connectivity leading to reduced controllability of brain networks (Zarkali et al., 2020b). These changes in functional connectivity include abnormal connections within the DMN, between posterior DMN regions and visual processing areas, or oscillations caused by excessive synchronization (Halje et al., 2019), leading to changes in brain metastability. Other studies propose that alterations in the brainstem’s sleep–wake and dream regulation, such as RBD leading to dream imagery intruding into the waking state (Diederich et al., 2009), and abnormal cortical-striatal reward processing, may be involved in the development of VHs (Garofalo et al., 2017). PDP patients also exhibit dysfunction in the ventral striatal dopamine transporter (Jaakkola et al., 2017), and the lack of dopamine activity affects retinal function, leading to abnormal “top-down” visual processing that replaces the normal bottom-up system (Archibald et al., 2009). This results in a failure to inhibit erroneous information or the spontaneous generation of internally created images through the pontine-geniculate-occipital (PGO) system (Diederich et al., 2005). The attentional control networks hypothesis suggests that hallucinations may arise from disruptions and overloads in attention networks, where new incoming stimuli are misclassified, leading to abnormal hierarchical processing and the intrusion of false perceptions into the stream of consciousness (Collerton et al., 2005). In addition, the thalamocortical dysrhythmia (TCD) DMN decoupling hypothesis posits that TCD-induced disturbances in high-order thalamic nucleus activity leads to a loss of fine-tuning in cortico-cortical regulation and the decoupling of the DMN (Onofrj et al., 2023). Changes in the dorsal attention network (DAN), ventral attention network (VAN), and DMN play crucial roles in the occurrence of MHs and VHs (Sawczak et al., 2019), with each network contributing to the perception and processing of information, adjustment of attention, and generation of selective attention (Zhong et al., 2021b). The underactivation of the VAN and overactivation of the DAN and DMN result in the incorrect recall of perceived information, leading to VHs (Shine et al., 2011). Dysfunction in the DAN is considered a key factor and early indicator of the progression from MHs to more widespread forms of hallucinations (Baik et al., 2024), suggesting the involvement of the basal forebrain and its broader impact on cortical cholinergic projections, particularly to the ventral occipitotemporal cortex. Thus, although labeled “minor,” MHs potentially serve as early biomarkers for the progression to fully-formed hallucinations (Bejr-Kasem et al., 2019).

Cortical changes can lead to neurotransmitter imbalances (Vignando et al., 2022), and current research supports the involvement of multiple neurotransmitter dysfunctions in the development of PDP. The keyword “5-HT2A inverse agonist(s)” reflects the role of the serotonin (5-HT) system in the mechanism of PDP. The involvement of the 5-HT system in PDP is supported by evidence that 5-HT agonists can induce delirium and psychosis, while reducing 5-HT activity can alleviate psychotic symptoms (van der Mast and Fekkes, 2000). In addition, the association between grey matter volume and the local expression of 5-HT1A and 5-HT2A receptor genes suggests that serotonergic receptors play a role in PDP (Pisani et al., 2023). Overactivation of 5-HT receptors, particularly the 5-HT2A receptor, is closely linked to hallucinations and delusions in Parkinson’s patients (Huot, 2018). This may occur through increased receptor binding in the inferior lateral temporal cortex (Huot et al., 2010), the prefrontal cortex, and the ventral visual pathways (Ballanger et al., 2010). Additionally, the activation of metabotropic glutamate receptor 2 (mGlu2) has been shown to reduce psychosis-like behaviors (PLBs) in 1-methyl-4-phenyl-1,2,3,6-tetrahydropyridine (MPTP)-lesioned macaques (Nuara et al., 2021). Beyond the 5-HT and mGlu2 systems, the dopamine system also plays a crucial role in PDP. The primary pathological feature of PD is the loss of dopaminergic neurons in the nigrostriatal pathway, leading to a decrease in dopamine levels in the striatum. However, despite the overall reduction in dopamine levels in Parkinson’s patients, the emergence of psychotic symptoms is often associated with hypersensitivity and imbalance in dopamine receptors (Seeman, 2006). The hypersensitivity of dopamine2 receptors is considered a key factor in PDP (Seeman, 2006; Seeman, 2011). This multifaceted interaction between neurotransmitter systems, particularly the serotonergic and dopaminergic pathways, underscores the complexity of PDP’s pathophysiology. These insights highlight the importance of targeted pharmacological interventions, such as 5-HT2A inverse agonists, to address the specific neurochemical imbalances contributing to PDP.

We observed an evolution of keywords from “dopaminergic psychosis” (e.g., levodopa, drug-induced psychosis, dopaminergic-induced hallucinations) and atypical antipsychotics (AAPs) (e.g., olanzapine, clozapine, risperidone) to “5-HT2A inverse agonist(s),” “pimavanserin,” and “Parkinson’s disease psychosis (PDP),” reflecting a shift in research focus from AAPs to novel anti-PDP drugs. Treating PDP poses a significant challenge due to the heterogeneity of its etiology, symptoms, and underlying mechanisms. Keywords like “risperidone,” “olanzapine,” and “clozapine” were prominent before 2007, while “pimavanserin” has become a current active topic. Retrospective database analyses have shown that olanzapine and risperidone are associated with an increased risk of mortality (Abdul-Rahman et al., 2024). Despite short-term benefits in improving Brief Psychiatric Rating Scale psychosis scores (Mohr et al., 2000), risperidone worsens UPDRS scores (Ellis et al., 2000). No clear benefits were observed with olanzapine (Wilby et al., 2017), and it had a higher incidence of adverse effects (Aarsland et al., 1999c). Therefore, interest in these drugs has declined in studies after 2007. Research has found that antagonizing the 5-HT2A receptor is the pharmacological basis for most PDP treatments. This mechanism likely reduces abnormally high-frequency oscillations in prefrontal structures and abnormal synchrony between different brain regions (Stan et al., 2024). Traditional antipsychotics like clozapine have shown some efficacy in controlling PDP symptoms due to their 5-HT2A receptor antagonism (Wilby et al., 2017). However, clozapine is now mainly used for emergencies or symptoms unresponsive to other treatments due to its side effect profile (Thames and Ondo, 2023). The evidence for quetiapine is mixed (Juncos et al., 2004; Wilby et al., 2017). Meanwhile, the 5-HT2A inverse agonist pimavanserin is considered a potential first-line alternative to clozapine (Srisurapanont et al., 2024). Among the top 10 cited papers, the second most cited is a clinical study related to pimavanserin, which has the highest impact factor and average annual citations.

Pimavanserin is the first and only AAPs approved by the FDA for the treatment of hallucinations and delusions associated with PDP. It exhibits selective inverse agonism at the 5-HT2A receptor, with greater affinity compared to the 5-HT2C receptor, and has no significant activity at any other G protein-coupled receptors (Hacksell et al., 2014). Patients treated with pimavanserin showed greater improvements in measures including SAPS and UPDRS Parts II and III (Meltzer et al., 2010), particularly in subgroups with more pronounced cognitive impairments, with the drug demonstrating good safety and tolerability (Espay et al., 2018; Isaacson et al., 2020). Pimavanserin has also been shown to improve nighttime sleep and daytime sleepiness (Patel et al., 2018), significantly reduce the risk of psychosis relapse in Parkinson’s patients with dementia (Weintraub et al., 2024), and does not worsen motor or cognitive functions (Alva et al., 2024). Additionally, it does not increase the risk of mortality (Ballard et al., 2020) and is not associated with excess death reports in FDA Adverse Event Reporting System (Brown et al., 2021). Compared to other AAPs, pimavanserin reduces the risk of falls or fractures in PDP patients (Layton et al., 2022) but may cause a slight prolongation of the QT interval (Torres-Yaghi et al., 2021). Monotherapy with pimavanserin is associated with lower all-cause hospitalization, psychiatric-related hospitalization rates (Rajagopalan et al., 2023a), and emergency department visit rates (Rajagopalan et al., 2023b), as well as reduced likelihood of all-cause healthcare resource use (Rajagopalan et al., 2024b) and risk of long-term care (LTC) admission (LTCA) (Rajagopalan et al., 2024a). The costs related to per-patient-per-year skilled nursing facility-stay and LTCA are also lower (Rajagopalan et al., 2024c).

Some 5-HT2A inverse agonists exhibit more potent effects than pimavanserin. Pimavanserin derivative 7–16 has shown higher 5-HT2A receptor antagonist activity, inverse agonist activity, and *in vivo* pharmacokinetic activity compared to pimavanserin. The efficacy window between 5-HT2A and hERG activities is increased, with good safety profiles (Ma et al., 2022). *In vitro* competitive receptor binding and functional G protein-coupled assays have demonstrated that compounds 2, 3, and 4 exhibit higher 5-HT2A inverse agonist potency in human cortical and recombinant cells than pimavanserin (Albujuq et al., 2023). LPM6690061 is a safe and effective 5-HT2A inverse agonist that shows significantly more potent antipsychotic-like effects than pimavanserin (Zhu et al., 2023). Nelotanserin, a 5-HT2A/2C inverse agonist, is being used to treat RBD and psychosis in Parkinson’s patients with dementia (Kwan et al., 2024). The potential efficacy of ulotaront (Kuvarzin et al., 2023), SYN-120 (Huot et al., 2017), mesdopetam (IRL790) (Stan et al., 2024), and EMD-281,014 (Hamadjida et al., 2018) in PDP has also been investigated.

Considering the potential ceiling effect of 5-HT2A receptor blockade (Huot, 2018), the mGlu2 pathway has become a research focus. The burst keyword “mptp-lesioned macaques” reflects the animal models in mGlu2 pathway-based research. The 5-HT2A receptor forms heterodimers with the mGlu2 receptor, producing equivalent downstream signaling effects through 5-HT2A receptor antagonism and mGlu2 activation. Activation of the mGlu2 receptor with mGluR2/3 orthosteric agonists (OAs) like LY-404,039 (Kang et al., 2023), LY-354,740 (Kwan et al., 2021a), and mGlu2-positive allosteric modulators (PAMs) like LY-487,379 (Sid-Otmane et al., 2020), CBiPES (Frouni et al., 2021), and biphenylindanone A (Kang et al., 2024) is effective in alleviating PLBs in MPTP-lesioned marmosets, while also enhancing the therapeutic benefits of levodopa (Frouni et al., 2019). Positive allosteric modulation of the mGlu2 receptor and orthosteric stimulation of mGlu2/3 receptors may represent a novel approach for treating L-3,4-dihydroxyphenylalanine (l-DOPA)-induced PDP, potentially enhancing the anti-PLB effects of 5-HT2A antagonism (Kwan et al., 2021a). Combining 5-HT2A receptor antagonism with mGluR2 activation results in a greater reduction of L-DOPA-induced PDP. A further additive effect can be achieved when an mGlu2-OA and an mGlu2-PAM are combined with a 5-HT2A receptor antagonist, compared to adding either an mGlu2-OA or an mGlu2-PAM alone to a 5-HT2A receptor antagonist. However, the combined use of mGlu2-OA and mGlu2-PAM does not yield greater therapeutic effects (Nuara et al., 2020). Future research may focus on developing mGlu2 agonists based on 5-HT2A antagonists.

The clustering labels “5-HT3 receptor antagonists” and “cholinesterase inhibitors” represent additional research directions. Selective blockade of the 5-HT3 receptor, such as with ondansetron, has been shown to alleviate psychosis in MPTP-lesioned marmosets (Kwan et al., 2021b) and reduce VHs and BPRS scores in PDP (Kwan and Huot, 2019). In addition, the effects of the acetylcholinesterase inhibitor carbatine on psychotic symptoms are not well established (Reilly et al., 2024) and may have a smaller effect size (d'Angremont et al., 2023), but it is considered a first-line treatment for mild VHs (Powell et al., 2022). Further research is needed to explore these areas.

In addition to pharmacological treatments, non-pharmacological therapies include ECT (Ueda et al., 2010), restoration of daytime lighting and nighttime sleep, behavioral interventions, and caregiver education (Pahwa et al., 2022). ECT has been shown to be effective in treating PDP (Rojas et al., 2022), particularly in patients with early-onset PD accompanied by psychosis (Yamaoka et al., 2022) and in cases of PD refractory psychosis (Ueda et al., 2010). Although large-scale randomized controlled trials are lacking, two case series reported improvements in PD psychosis spectrum symptoms with ECT. After 5–12 sessions of ECT, SAPS scores improved within 1 week (Usui et al., 2011), with effects in some patients lasting 5–30 weeks after 6 or more sessions (Ueda et al., 2010). Additionally, unilateral anterior cingulotomy combined with bilateral STN-DBS has been shown to improve PDP (Wang et al., 2024).

The keyword “nursing home placement” highlights the significant role of LTC facilities and caregivers in the treatment strategies for PDP. The uncontrollable symptoms in PDP patients often necessitate their transfer to nursing homes, as they face increased risks, including mortality (Kang and Bronstein, 2004). Caregivers play a crucial role in the daily care and psychological support of PDP patients, but they themselves are under tremendous psychological and physical stress (Mantri et al., 2021b), including the challenge of communicating with healthcare institutions and professionals (Mantri et al., 2021a). Moreover, hallucinations in PDP patients significantly impact caregivers’ quality of life (Powell et al., 2022). Therefore, implementing proactive intervention strategies to prevent, delay, or mitigate the severity of PDP is essential for improving caregivers’ quality of life (Fredericks et al., 2017). Studies have shown that care partners prefer non-pharmacological management strategies and have a higher demand for access to medical information (Mantri et al., 2021a). Telemedicine is viewed as a means to enhance the care provided by specialists and LTC staff for PDP patients (Shaughnessy et al., 2022). The burden on caregivers not only affects their health but also directly impacts the care quality and life quality of the patients (Schaffer and Henry, 2023). Therefore, providing emotional and informational support to caregivers is crucial (Shafer et al., 2023). In recent years, support programs and interventions for caregivers, such as psychological counseling, social support networks, educational training, and caregiver support groups, have increasingly become a focus of research (Friedman and Kennedy, 2021).

The recently burst keyword “risk” highlights the risk factors associated with PDP and the challenges in its treatment. Factors such as age, disease duration, motor symptoms, sleep quality, health-related quality of life (Zhong et al., 2021a), excessive daytime sleepiness, autonomic symptom burden, and the density of Cholinergic nucleus 4 (Barrett et al., 2018) have been associated with MHs. Late-stage Hoehn-Yahr classification and frontal lobe dysfunction are considered independent risk factors for MHs (Zhang et al., 2021). Additionally, MHs (Schneider et al., 2024), current smoking status (Terravecchia et al., 2021), cognitive impairment (Lenka et al., 2023), anxiety (Factor et al., 2017), gastrointestinal autonomic dysfunction, RBD, the Aβ42/total tau ratio (Chen et al., 2024), and grey matter volume within specific structural covariance networks (Knolle et al., 2023) are independent predictors of PDP. Monitoring and managing these risk factors can aid in the early identification and delay the onset of PDP.

Studies have shown that over one-third of Parkinson’s patients discontinue antipsychotic treatment (Pham Nguyen et al., 2021). Factors such as younger age, male gender, anemia, use of anti-anxiety medication or anxiety, use of sedatives/hypnotics, bladder disease, coronary artery disease, diabetes, hypertension, and dementia are more likely associated with changes in PDP treatment in LTC settings (Rashid et al., 2022). Low doses of AAPs are commonly prescribed off-label. Therefore, for patients who cannot tolerate quetiapine or clozapine, a gradual transition to pimavanserin while maintaining adequate 5-HT2A antagonism is recommended (Black et al., 2018). Integrating clinical pharmacists into outpatient neurology clinics can improve the accessibility of pimavanserin (Livezey et al., 2021), helping maintain treatment adherence and reduce risks.

This study was the first to explore the development trends and potential frontiers of PDP over the past few decades from a bibliometric perspective. It included 603 articles across 54 countries, 207 journals, and 857 institutions. The analysis covered annual publication volumes, prolific countries, journals, institutions, as well as highly cited articles and burst keywords, providing a wealth of data. Additionally, we used CiteSpace and VOSviewer to transform quantitative literature data into visual maps and networks, making the information more accessible. However, this study has some limitations. According to CiteSpace formatting requirements, we included only review and article formats, which may lead to an incomplete representation of relevant publications. Articles published after the search date were not included, potentially affecting the timeliness of the research.

While our current analysis primarily focuses on bibliometric output, future studies could explore the relationship between research funding and publication output in the field of PDP. Understanding how funding correlates with output may reveal important insights into global research trends. For example, data from funding agencies such as the National Institutes of Health or Horizon Europe could be correlated with bibliographic data to assess the impact of financial investment on research outcomes in PDP.

## 5 Conclusion

Research on PDP is advancing rapidly and showing promising trends. Many countries, institutions, and authors have contributed to this field. Topics such as brain, prevalence, connectivity, and AAPs have received extensive attention. Current research hotspots include functional connectivity, pimavanserin, and risk factors. In the field of new drug development, novel 5-HT2A receptor inverse agonists and mGlu2 agonists based on 5-HT2A antagonists are being extensively studied. However, there is low connectivity between countries. Future efforts should focus on strengthening international academic collaborations around PDP research hotspots to enhance the impact of this field and increase the publication of highly cited articles.

## Data Availability

The original contributions presented in the study are included in the article/supplementary material, further inquiries can be directed to the corresponding authors.
